# Turning Potential Into Action: Using Pluripotent Stem Cells to Understand Heart Development and Function in Health and Disease

**DOI:** 10.1002/sctm.16-0476

**Published:** 2017-03-24

**Authors:** Hananeh Fonoudi, Alexis Bosman

**Affiliations:** ^1^ Developmental and Stem Cell Biology Division Victor Chang Cardiac Research Institute Sydney Australia; ^2^ Griffith University School of Medicine Gold Coast Queensland Australia

## Abstract

Pluripotent stem cells hold enormous potential for regenerative therapies, however their ability to provide insight into early human development and the origins of disease could arguably provide an even greater outcome. This is primarily due to their contribution to the establishment of a powerful knowledge base of human development, something which all researchers and clinicians can potentially benefit from. Modeling human heart development and disease using pluripotent stem cells has already provided many important insights into cardiogenesis and cardiovascular disease mechanisms however, it is important to be aware of the complexities of this model system. Thorough contemplation of experimental models and specialized techniques is required to provide high‐quality evidence of the intricacies of both normal early development, and when this process goes awry in disease states. Stem Cells Translational Medicine *2017;6:1452–1457*

**Significance Statement**

This Perspective article provides a brief overview of the current and potential uses of pluripotent stem cells for investigating early development of the human heart in both health and in disease states. Additionally, it provides guidance and insight into the complexities of establishing pluripotent stem cell models to probe early cardiogenesis and cardiovascular disease.

“Heart and Brain are the two lords of life. In the metaphors of ordinary speech and in the stricter language of science, we use these terms to indicate two central powers, from which all motives radiate, to which all influences converge.”

— George Henry Lewes

## Introduction

Understanding early human development is a complicated and technically difficult process. The formation of a complex biological organism such as a human is the result of a plethora of intricate cellular mechanisms and biochemical processes interacting in time and space to form a highly sophisticated entity with multiple systems, organs, and intermediate structures. Further, understanding the development of the heart is of great interest not only for developmental biologists, but also for those studying diseases resulting in structural or functional cardiovascular deficits, and those treating individuals with these deficits. According to the World Health Organization, cardiovascular disease is the leading cause of death globally 1, therefore it is imperative we increase our understanding of how the heart develops and functions, both under normal physiological conditions, as well as in diseased states.

When attempting to examine organogenesis in humans, it is particularly difficult to uncover the pathways involved in this complex process due to technical difficulties and ethical concerns associated with human‐based research. Further, because of these limitations there is—justifiably—restricted availability of human tissue available for scientific purposes, even though access to this material would help to unlock the secrets of early development. In 1981, a discovery was made which changed the way we looked at mammalian development‐pluripotent cells were “born.” First discovered as embryonic stem cells in mouse 2, this was then controversially mimicked in humans 3, and heralded a new era in developmental biology. However, as they say, necessity is the mother of invention, and pluripotent stem cells were then sensationally reinvented in 2007 as the universally ethically acceptable induced pluripotent stem cells (iPSC) 4. The appeal of pluripotent stem cells (PSC) was not lost on any who understood their potential—cells which have the infinite capability to produce the plethora of cell types found in the human body. Since then, the concept of a “make‐your‐own cure” to degenerative diseases has been the twinkle in the eye of the public, clinicians and stem cell researchers alike. It is important however, to realize the even greater potential of these cells—uncovering the intricacies of human development to create a developmental knowledge base for all to benefit from. Therefore, human PSC (hPSC) have become a powerful tool in allowing researchers to examine early human development, circumventing the need for primary tissues 5, 6, 7. In this Perspective article, we will briefly discuss what is known about normal heart development and the different factors involved in early cardiogenesis and how hPSC can help us to better understand this process. Current in vitro uses for hPSC‐CMs are addressed along with the clinical applications of these cells. Finally, some practicalities of working with these cells are shared in order to give insight to the intricacies of their successful use.

## Pluripotent Stem Cells Helping to Unravel Heart Development

The heart is a very complex organ. The cellular bulk of heart tissue mainly consists of cardiomyocytes and fibroblasts however these are not the only cell types which play an important role in cardiac development and function. Cells of the conduction system (nodal, bundle of His, bundle branch and Purkinje cells), inflammatory cells, blood vessels, endocardial and epicardial cells must also be considered. All these cell types play an important role in the correct functioning of the heart, and the body as a whole. The heart responds to sympathetic/parasympathetic stimulation—for example through psychological stress or increases/reductions in blood pressure and volume—as well as to pain, ischemia, oxygen concentration variation, changes in blood flow, and chemical/pharmacological insult.

The heart is the first functional organ created during mammalian embryonic development, derived from the mesodermal germ layer established in the early embryo. There are two distinct heart fields which contribute to the formation of the intricately complex four‐chambered heart in a spatially and temporally defined manner. Upon gastrulation, the primitive streak is formed consisting of a layer of cells that establish the embryonic midline. In second week of human gestation, mesenchymal cells migrate through the streak and move out to create heart‐forming regions. By week 3, this crescent shaped mesodermal region, often called the cardiac crescent, expands to form a primitive heart tube, made up of an exterior layer of myocardial cells and an interior layer of endocardial cells, separated by an extracellular matrix 8. The heartbeat is initiated around this stage, followed by the progression of numerous cell divisions to form the common atrium and ventricles. The electrocardiogram also becomes clear at this stage, where it can be observed that cells of the primary heart tube possess low contractility and velocity, as opposed to cells of the chamber myocardium, which have high contractility and velocity 9, indicating early divergent identities of cardiomyocytes in regards to their electrophysiological properties in the early heart.

The “transcriptional machinery” of the heart is made up of an evolutionarily conserved group of transcription factors that reinforce one another's expression and can either act singularly, or in conjunction, for the expression of genes required for proper cardiac development 10. Signaling pathways associated with early cardiac development include the bone morphogenic protein, Wnt and fibroblast growth factor signaling pathways. Also associated are sonic hedgehog (Shh) and Notch proteins.

**Figure 1:**
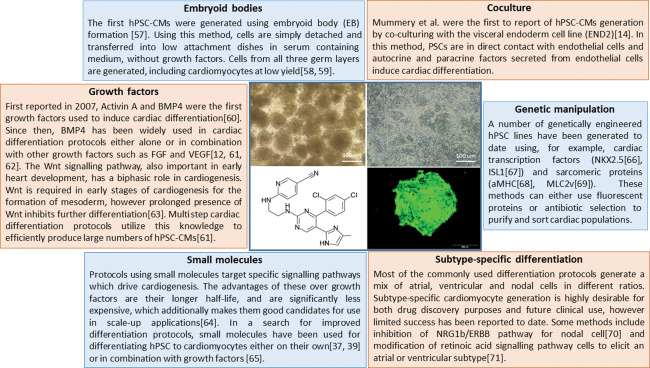
Summary of different methods used to differentiate hPSCs to cardiomyocytes.

The concept of inducing cardiogenesis in PSC is to attempt to mimic the normal development of the heart in a dish. In vitro differentiation of PSCs into cardiomyocytes can be broken down to three critical steps; (a) mesoendodermal differentiation, (b) mesoderm generation, and (c) cardiac specification. These three processes can, and have been, achieved by combining different cytokines and small molecules in a tightly time‐ and dose‐dependent manner 11. Cardiac specification can also be influenced by the manipulation of the microenvironment, such as using matrices 12, carrier proteins 13 or coculturing with specific cell types 14. This is further described in Figure 1.

Although hPSCs can be differentiated to CMs, so far it has been shown that they are relatively immature and maintain fetal characteristics. Efforts toward maturing these cells in vitro will help to further understand how functional and developmental characteristics change over time.

## Current In Vitro Uses for PSC‐Derived Cardiac Cells

The various uses of hPSC to date have included: modeling disease with known mutations 15; creating cells for drug testing purposes 16, 17; understanding the concept of cell identity and potential 18; creating cell‐specific populations for possible use in regenerative models of disease 19, 20, and of course, examining both normal early human development 7 and in diseased states 21. Some more creative uses of these cells have also been suggested, one being the use of hPSC‐derived cardiomyocytes (hPSC‐CMs) for making microbiomachines 22. Here, we will focus on the disease modeling applications of these cells.

The use of hPSC‐CMs for modeling disease has shown to be fruitful not only in monogenic diseases, but also for complex diseases such as trisomies and syndromes 21, where the problem of penetrance or individual disease variations could have theoretically stymied their successful use. An extremely important factor in effectively modeling complex disease is ensuring all controls used are appropriate (e.g., related individuals, generated in the same laboratory in the same manner, identical culture conditions, etc.) 23. This is even more crucial when the target disease is highly heterogeneous, or when only subtle differences between patients and controls are observed. The confounding factors which may affect the experimental outcomes include; the origin of primary reprogrammed cell, reprogramming method, genetic background, age, sex, comorbidities, and so forth. Therefore, a superior experimental design would be one which, beside the disease phenotype, has the most similarity between patients and controls. Additionally, having multiple clones from the same individual helps to eliminate the effects of reprogramming itself on the cells and any heterogeneity ensuing from the experimental processes performed.

Further bolstering models of heterogenous disease is to make a cohort of numerous patients, which ensures a more robust outcome and confirms that specific observed phenotypes are not merely due to genetic characteristics of individual patients. In the case of complex diseases when multiple unknown genetic loci are involved, patient‐derived iPSC or diseased embryonic stem cell lines are the best option. On the other hand, when the effect of a single genetic variation is questioned irrespective of the genetic background, genome editing, such as with CRISPR/Cas9 24, 25, in unaffected PSC is the preferred model. It is worth mentioning that current genetic manipulation techniques are not perfect. Generation of edited cell lines is usually a time‐consuming process, and the CRISPR/Cas9 system can have off‐target effects. However, the technology in this area is developing rapidly and systems with greater efficiency and less off‐targets have been introduced which will soon facilitate the creation of isogenic models of disease 26, 27.

## The Clinical Landscape of PSC‐Derived Cardiac Cells

Heart failure places an enormous burden on health systems worldwide. Patients suffering from heart failure have a poor 5‐year mortality rate of approximately 50% 28 with limited treatment options. Currently, the best treatment for heart failure is transplantation, which for the majority of patients, is not a likely reality. The main cause of heart failure is ischemic heart disease 29, however congenital diseases, such as hypoplastic left heart syndrome and transposition of the great arteries, should also be considered in this burden, even though the number of affected individuals is comparatively small.

Preclinical trials using hPSC‐CMs in non‐human primates and porcine models have shown some promise for the treatment of heart failure 29, 30, 31, and although hPSC‐CMs therapies have yet to be rigorously tested in humans, there has been evidence that other stem cell types may provide some benefit. Therapeutic trials using cardiac stem cells, mesenchymal stem cells, and bone marrow‐derived cells have variably been shown to improve certain endpoints, however their true outcomes remain controversial 32, 33. Improvements in these models are suspected to be a consequence of paracrine functions of the transplanted cells and not due to differentiation into cardiomyocytes, however this has not yet been definitively ascertained 28. The first trial of hPSC‐derived cardiac progenitor cells involving a single post‐myocardial infarction patient was performed in 2015 34, providing evidence of the feasibility of using hPSC for treatment of heart failure. Much work is still required to discover if this will be a promising option for treatments in the future.

For stem cell therapies to work, it is postulated that transplanted cells should be able to fully integrate and mature into cells similar to those found in vivo 29. One school of thought suggests that transplantation of cells similar to the native tissue should be used 29, whereas others propose an earlier or progenitor type cell be transplanted to allow the cells opportunity to adapt to the native tissue environment 34. It will only be through rigorous high‐quality studies that this will eventually be ascertained.

An important step for the clinical success of hPSC for regenerative medicine is being able to reproducibly generate large numbers of highly‐purified cells under clinical good manufacturing practice conditions 35, 36. Although many articles have reported on ways to produce highly pure cardiomyocyte populations, the success of these methods has been variable from line to line. The reality is that every individual stem cell line is different, and thus responds differently to differentiation protocols, even in experienced hands and when well defined 11. To date, it has been extremely difficult to reproducibly create functional cells with highly‐purified populations from numerous genetically unique lines 37, 38, 39. The largest study done to date using a small molecule‐based cardiac differentiation protocol reported high reproducibility on a record 23 genetically individual pluripotent stem cell lines (51 total lines, including clones) 40. Possible clinical applications of hPSC‐CMs will be hindered by this huge roadblock in the regenerative medicine field if personalized therapies are the way forward to treatment.

## Practicalities of Making hPSC‐CMs

The laboratory environment is one that is highly contrived and obviously not precisely mimicking normal development. Although some excellent methods have been published on how to efficiently differentiate hPSC into cardiomyocytes, how these actually represent in vivo cells is still unanswered. It is known however, that the maturity of these cells is limited to very early developmental stages 41. It also has been shown that hPSC‐CMs are smaller when compared to adult human cardiomyocytes. Cell size is very important because it affects the membrane capacitance, contractile force and action potential depolarization 42. During cardiac maturation, electrophysiology, mitochondrial content, expression of cardiac specific isoforms, and sarcomere organization changes, which will consequently change the functionality of the cardiomyocytes. So far, different approaches have been used to enhance hPSC‐CMs maturation levels, such as long term culture 43, substrate stiffness 44, cell patterning approaches 45, 46, electric stimulation 47, 48, or addition of different biochemical materials such as adrenergic receptor agonists 49 and Triiodothyronine 50. However, the problem with these methods is that they are usually time consuming and by themselves have only resulted to an intermediate state of maturation. The best result achieved so far is to neonatal stage. This area is currently the focus of a large number of studies which have the aim to produce more mature type cells. One possible approach would be to combine two or more of the maturation methods in order to enhance the final outcome.

When considering hPSC‐CMs and their role in modeling both development and cardiac disease, one must not only consider the role of the cell functionally, but also the role of cell‐to‐cell interactions (e.g., intercalated discs, gap junctions, desmosomes), and the environment in which the cells reside, (e.g., extracellular matrix, three‐dimensional [3D] spatiality, shear forces). Given the complexity of mechanical, structural and hemodynamic interactions in normal development, it is challenging to isolate specific signals which may affect development and remodeling responses in vivo, particularly since stretch and strain often vary in vitro 51. Therefore, existing experimental techniques still remain inadequate in uncovering differences between diseased versus nondiseased, and in vivo and in vitro produced cells. This however, should not preclude the use of these cells in complex models of cardiac disease and development, as many undiscovered or surprising outcomes have still been revealed using current techniques 21.

**Figure 2:**
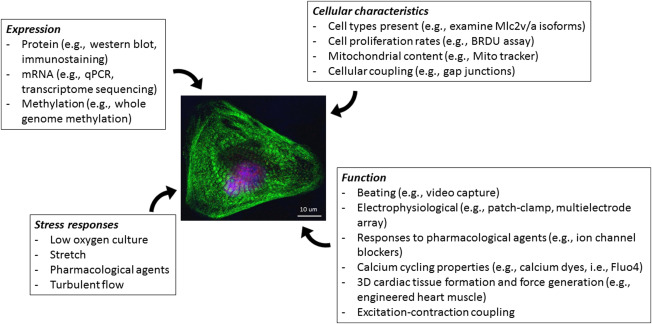
Experimental procedures to consider when performing experiments with PSC‐CMs. **Expression**: hPSC‐CMs can be analyzed for the expression of proteins, RNA, methylation markings. **Cellular characteristics**: These methods used to examine the physical characteristics of cells will give insight into many types of diseases, particularly those with functional consequences. **Function**: The most challenging aspect of using hPSC‐CM is assessing their function. Methods included are specialized and require expert advice. For example, engineered heart muscle is a 3D tissue construct made of mixture of cardiomyocytes, fibroblasts and extracellular matrix which can be formed within ring shaped molds, embedded with rigid posts, or elongated into rigid mesh. These can be used to measure the force of contraction of PSC‐CM, and has also been shown to induce sarcomere assembly and maturation. Patch clamping is the gold standard method for the electrophysiological characterization of cardiomyocytes. Using this method, functionality of the cells, maturity level and even subtype of the cells can be revealed. This method, however is extremely time consuming and low‐throughput. Multielectrode array (MEA) is a non‐invasive method for detection of field potential.This method can be used for high‐throughput safety screening of drugs. Excitation‐contraction coupling can be investigated using dye transfer techniques. **Stress responses**: Cellular responses to external stress, such as culturing cells in low oxygen level and stretch, can also be used to probe the functionality of hPSC‐CMs characteristics. (see References 41, 52, 54, 55, 56, 57, 73, 74, 75)

If making one or one million cardiomyocytes, it is important to remember that this cell is merely one building block of a larger, more complex organ. One must consider the outcome of what any experiment is set up to achieve (Fig. 2). The heart is not merely an electrically stimulated muscular pump for the blood, but a neurally integrated, hemodynamically active organ which responds to changes in the body as a whole, compensating homeostatically to changes in blood pressure, stress, and access to nutrients and oxygen. The importance of the 3D structure of the heart and each cell's contribution to the organ must be also acknowledged, and finally the importance of cell coupling—both electrically and physically—must also be considered. The development of engineered tissues 52 or organoids 53 now give us the opportunity to examine interactions between different cell types and now have an even better understanding of what may be occurring physiologically in the heart. Engineered heart muscle is a 3D tissue construct made of mixture of cardiomyocytes, fibroblasts and extracellular matrix which can be formed within ring shaped molds 52, embedded with rigid posts 54, 55, or elongated into rigid mesh 41, 56. These can be used to measure the force of contraction of hPSC‐CMs, and has also been shown to induce sarcomere assembly and maturation 57. To make these tissues requires more than a million cells, which limits greater applications of these structures. A current aim in this sphere is to miniaturize this system in order to facilitate their mass production, and thus, enhance their application in disease modeling and drug screening platforms 57. With the plethora of uses these versatile cells provide, it is exciting to consider what might be the next step in this already electrified field of research.

## Author Contributions

H.F. and A.B.: manuscript writing, final approval of the manuscript.

## Disclosure of Potential Conflicts of Interest

The authors indicated no potential conflicts of interest.
